# Characterization and Fungicide Sensitivity of *Phaeosphaeriopsis obtusispora* That Causes Marginal Leaf Blight in *Agave hybrid* H.11648

**DOI:** 10.3390/jof10070486

**Published:** 2024-07-14

**Authors:** Weihuai Wu, Guihua Wang, Erli Li, Shibei Tan, Gang Xu, Xing Huang, Helong Chen, Yanqiong Liang, Rui Li, Jianfeng Qin, Kexian Yi

**Affiliations:** 1 Hainan Key Laboratory for Monitoring and Control of Tropical Agricultural Pests, Environment and Plant Protection Institute, Chinese Academy of Tropical Agricultural Sciences, Haikou 571101, China; wgh8308@163.com (G.W.); tanshibei915@163.com (S.T.); xugangxiwang@163.com (G.X.); chenhelong951@126.com (H.C.); yanqiongliang@126.com (Y.L.); ruili202305@163.com (R.L.); 2 School of Tropical Agriculture and Forestry, Hainan University, Haikou 570228, China; 3 Guangxi Subtropical Crops Research Institute, Nanning 530001, China; qinjianfengmail@163.com; 4 Sanya Research Institute, Chinese Academy of Tropical Agricultural Sciences, Sanya 572025, China

**Keywords:** sisal, fungal disease, pathogen identification, biological characteristics

## Abstract

Sisal is an important tropical cash crop in southern China. Unfortunately, it is threatened by various diseases. In 2022, a new disease tentatively named marginal leaf blight disease (MLBD) was first observed in sisal fields across Guangxi and Guangdong provinces, with an incidence rate ranging from 13% to 30%. In this work, to isolate and identify the pathogens causing MLBD, sisal leaves exhibiting the typical MLBD symptoms were collected, and nine strains were obtained. Pathogenicity tests, morphological observations, and phylogenetic analyses confirmed that two strains, namely 22GX1-3 and 22GD1-4, identified as *Phaeosphaeriopsis obtusispora*, were the causative pathogens of MLBD. Further investigations into the biological characteristics of *P. obtusispora* showed that its mycelia exhibited optimal growth on PDA medium, with the most favourable temperature and pH being 25 °C and 7.0, respectively. The mycelia could grow in temperatures ranging from 10 °C to 32 °C but ceased at 35 °C. Lactose and yeast extract powder were also identified as the optimal carbon and nitrogen sources, respectively. Additionally, the effectiveness of various control agents was assessed on a single strain, 22GX1-3. Among the twelve fungicides tested, difenoconazole was proven the most effective, with an EC_50_ value of 0.5045 µg/mL. To our knowledge, this is the first report for sisal MLBD caused by *P. obtusispora*. Our results provide crucial pieces of information for the development of effective management strategies to control sisal MLBD caused by *P. obtusispora*.

## 1. Introduction

Sisal (*Agave* spp.) is a perennial monocotyledonous succulent plant belonging to the family Asparagaceae [1]. It has been extensively cultivated as an important tropical hard fiber crop in more than twenty countries or regions across Africa, Asia, and North and South America [1,2]. Globally, several *Agave* cultivars, such as *Agave sisalana* (Sisal), *Agave fourcroydes* (henequen), and hybrid 11648 ((*A. amaniensis* × *A. angustifolia*) × *A. amaniensis*), are suitable for fiber production. Nonetheless, only the *Agave hybrid* H.11648 has been commercially exploited due to its superior productivity and robustness [3]. *Agave* hybrid H.11648 was first introduced from Tanzania to China in 1963 [4]. To date, China has been one of the top ten producing countries for sisal fibre in the world, comprising 18,667 ha of cultivated area in Guangxi (81%), Guangdong (15%), and Hainan (4%) provinces, and accounting for more than 28% of the production worldwide [5]. In recent years, the cultivation of sisal has proven instrumental in elevating farmers’ incomes and fostering economic development, particularly in the impoverished regions of southwestern China [6].

As sisal cultivation has become more widespread, numerous diseases have emerged, including zebra disease [7], black spot disease [8], purple leafroll disease [4,9], and leaf blight disease [5]. These diseases pose a significant threat to the development of the sisal industry in China. In January 2022, a disease tentatively identified as marginal leaf blight disease (MLBD) was first discovered in the *Agave hybrid* H.11648 at Hongshan Farm in Guangxi Province and Dongfanghong Farm in Guangdong Province, China [10]. This disease is characterized by widespread black or reddish-brown lesions on the leaf apex and leaf margin, resulting in the formation of withered and curled leaves. Field investigation demonstrated that generally 13% to 20% of plants were infected by this disease. Serious disease outbreaks can cause infection in nearly 30% of plants. Although the disease will not result in plant death, it will induce a significant reduction in the production and quality of sisal fiber.

The identification of plant pathogens, the biological characterization, and the fungicide screening of the pathogen are crucial for the effective control of fungal plant diseases. Currently, pathogen isolation, Koch’s postulates, morphological observation, and molecular identification are the main means of plant pathogen diagnosis [11,12]. Temperature, pH, carbon source, and nitrogen source are the main factors affecting the growth of pathogenic fungi [13,14]. Triazole, phenylaminopyrimidine, and methoxyacrylate are the common fungicides used to prevent and control fungal diseases [15,16,17,18,19,20]. In this study, by performing symptom analysis, it was found that MLBD symptoms differ from those of other diseases involving sisal [5,6,7,8,9], suggesting they could be ascribed to a new disease. To effectively prevent and control the disease, the following objectives were set: (i) to isolate, test pathogenicity and identify pathogens that cause MLBD, (ii) to determine the biological characterization, and (iii) to evaluate the sensitivity of this pathogen to common fungicides to provide a basis for the recognition and prevention of this disease.

## 2. Materials and Methods

### 2.1. Collection and Isolation of Samples

**Figure 1:**
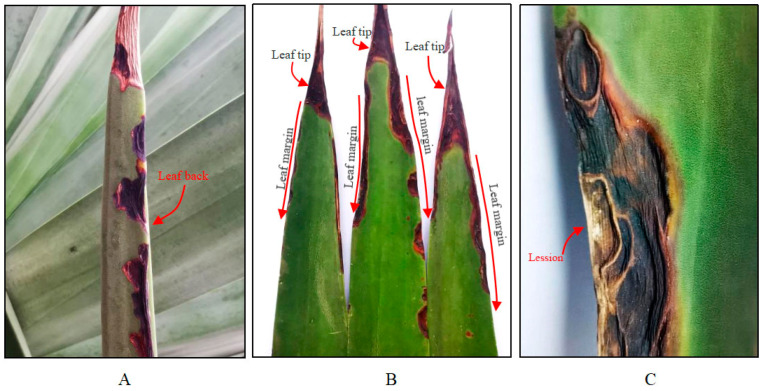
Field symptoms of sisal marginal leaf blight disease (SMLB). (**A**). Symptoms on the abaxial leaf. (**B**). Symptoms on the adaxial leaves. (**C**). Typical symptom of SMLB.

In January 2022, six leaves showing typical MLBD symptoms (Figure 1) were collected from two sisal plantations: three samples from Hongshan Farm in Guangxi Province and three samples from Dongfanghong Farm in Guangdong Province. The leaf samples showing disease symptoms were cut into 5 × 5 mm^2^ segments with the lesioned areas. The segments were successively soaked in 75% ethanol for 10–20 s, 0.1% mercuric chloride solution for 30 s, and washed in sterile water three times. Then, the segments were air-dried and cultured on potato dextrose agar (PDA) (Solarbio, Beijing, China) at 28 ± 1 °C for 5 to 7 d until colony formation. The hyphal edges of developing colonies, which varied in colour, texture, and other morphological characteristics, were transferred to PDA and cultured at 28 ± 1 °C with a 12-hour photoperiod for 5 d. After three rounds of re-isolation and re-cultivation, pure colonies exhibiting diverse appearances were obtained. The strains were stored in a refrigerator at 4 °C. All strains were stored and maintained at the Chinese Academy of Tropical Agricultural Sciences.

### 2.2. Pathogenicity Determination

The pathogenicity of all fungal strains obtained from sisal leaves exhibiting the typical MLBD symptoms was assessed on healthly 10-month-old sisal walking stem seedlings (5–8 leaves). These seedlings were collected from healthy sisal fields and cultivated in the greenhouse by regular water and fertilizer management until needed. Before inoculation, a target leaf of each plant was rinsed with sterile distilled water several times, air-dried, and artificially wounded with a sterile needle four to eight times at each inoculation point. Each target leaf had three inoculation points. Then, these target points were subjected to inoculation with mycelial plugs (5 mm in diameter) (three plants) or conidial suspensions (1 × 10^−6^ spores·mL^−1^) (three plants) using all the fungal strains. The conidial suspension was obtained by washing conidia with sterile distilled water and adjusting to 1 × 10^6^ spores·mL^−1^. An equal number of inoculation points from healthy plants were inoculated with sterile PDA plugs (5 mm in diameter) or sprayed with distilled water as controls. Fungal strains designated for mycelial plug inoculations were cultured on PDA plates for 10 d, while those for conidial suspension inoculations were grown for 18 d on PDA plates. All the inoculated leaves were enclosed in plastic bags to maintain high relative humidity, which were removed after 48 h. Subsequently, the plants were kept in a growth chamber set at 28 °C with 60%–70% humidity and monitored for symptoms daily. Upon the appearance of symptoms, the fungus was reisolated and identified through its morphological and molecular traits to fulfill Koch’s postulates.

### 2.3. Morphological Observations of Pathogens

The fungal pathogens were cultured on PDA in a climate chamber (12 h light/12 h dark) at 28 ± 1 °C for 15 d. Cultural features, including the colony’s upper and lower surfaces, texture, and colour, were observed. The fungal pathogens were cultured on PDA at 28 ± 1 °C for 30 d until spores formed. A small amount of the spores and mycelia were picked using a sterile needle and placed on a glass slide with a few drops of water for examination. The morphological features of conidiophores and spores, including the dimensions and form, were observed under a light microscope (Nikon, Tokyo, Japan). A hundred measurements for each spore were recorded using an ocular micrometer. To view the details of mature conidia ultrastructures, electron microscopy measurements were performed using a TM4000 Plus (Hitachi Ltd., Tokyo, Japan) scanning electron microscope. Particularly, culture mediums with sporulating mycelial blocks were cut into small pieces of ~3 mm^3^, immersed in a 2.5% glutaraldehyde solution (pH 7.2), and placed in a refrigerator at 4 °C for 12 h. After fixation, the samples were rinsed with 0.1% PBS four times and dehydrated consecutively with 30, 50, 70, 90, and 100% ethanol for 15–20 min each time. After dehydration, the ethanol was replaced with tertiary butanol and the samples were placed in a critical point drier, immersed in liquid carbon dioxide, and heated to above the critical temperature point of 31.4 °C to dry by evaporation. After drying, the ultrastructures of the mature conidia were observed using a TM4000 Plus (Hitachi Ltd., Tokyo, Japan) scanning electron microscope [21].

### 2.4. DNA Extraction and Multiple Sequence Analysis of Pathogens

The fungal genomic DNA was extracted from mycelia cultured on PDA for 10 d using a Fungal DNA Extraction Kit (OMEGA, Beijing, China), according to the manufacturer’s instructions. The internal transcribed spacer (ITS) sequence was amplified using the primers ITS1 (5′-TCCGTAGGTGAACCTGCGG-3′)/ITS4 (5′-TCCTCCGCTTAT TGATATGC-3′) [22] from the total genomic DNA of each strain. The primers LROR(F) (5′-ACCCGCTGAACTTAAGC-3′)/LR5(R)(5′−TCCTGAGGGAAACTTCG−3′) [23] were used to amplify the large subunit ribosomal gene (LSU) sequence. The primers fRPB2-5F (F) (5′−GAYGAYMGWGATCAYTTYGG−3′)/fRPB2-7cR (R)(5′−CCCATRGCTTGTYYR CCCAT−3′) [24] were employed to amplify the second largest RNA polymerase subunit (*RPB2*) sequence.

The PCR reaction mixture volume was 25 µL: 12.5 µL of 2  × Taq PCR Master Mix (Tiangen Biotech Co., Ltd., Beijing, China), 1 µL of each primer, 1 µL of DNA template, and 9.5 µL of RNase-free water. The PCR reaction program was as follows: pre-denaturation at 94 °C for 2 min; 32 cycles of denaturation at 94 °C for 50 s, annealing at 55 °C for 15 s, and extension at 72 °C for 3 min; and a final extension at 72 °C for 10 min. The amplified products were detected using 1% agarose gel electrophoresis, cloned, and sequenced. DNA sequence homology searches were performed using online BLAST searches of GenBank (Table S1) (5 May 2024), and the corresponding ITS regions, *LUS*, and *RPB2* sequences were downloaded. A multiple sequence alignment was performed using DNASTAR (DNAStar, Madison, WI, USA). The aligned sequences were concatenated using the concatenate sequence tool after the trimAI modification [25]. A phylogenetic tree was also constructed using the neighbour-joining method in MEGA 11.0. *Didymella exigua* CBS 183.55 was used as the outgroup, and branch support was evaluated using 1000 bootstrap replicates.

### 2.5. Determination of the Optimal Growth Conditions of the Pathogen

The mycelial plugs used in this work were all cut from the margins of the pathogenic fungal colonies that had grown for 10 d on PDA and achieved diameters of 5 mm. To characterize the impact of different media on mycelial growth, the mycelial plugs were inoculated onto PDA, PSA, Czapek, OMA, CMA, WA, and BMP media (Table S2) and cultured at 28 °C. To clarify the influence of the temperature on mycelial growth, mycelial plugs were inoculated in the center of the PDA and cultured in constant temperature incubators set at 10 °C, 15 °C, 20 °C, 25 °C, 28 °C, 30 °C, 32 °C, and 35 °C. To clarify the impact of carbon and nitrogen sources on mycelial growth, Czapek medium with maltose, fructose, glucose, lactose, mannitol, soluble starch, xylose, or D-sorbitol as the sole carbon source was used. Moreover, Czapek media with different nitrogen sources were produced using equal amounts of ammonium chloride, peptone, yeast extract powder, urea, beef extract, glycine, isoleucine, leucine or ammonium sulfate as the sole nitrogen source. The mycelial plugs were inoculated on the center of the Czapek medium and cultured at 28 °C. To investigate the impact of pH on mycelial growth, 1 mol L^−1^ HCl and NaOH were used to adjust the pH of the PDA to 3, 4, 5, 6, 7, 8, 9, 10, 11, and 12. Mycelial plugs were then inoculated on the PDA at various pH levels and cultured at 28 °C. Each strain was incubated at a temperature of 28 ± 1 °C for 30 d and the experiment was conducted using three replicates. The diameters of the colonies were measured using the cross method.

### 2.6. Sensitivity of Mycelial Growth to Twelve Fungicides

The inhibitory activities of twelve fungicides against pathogens were determined using the mycelial growth rate method. Three different types of fungicides, including triazole fungicides [difenoconazole (96% a.i.), tebuconazole (97.1% a.i.), myclobutanil (96% a.i.), thiophanate-methyl (95% a.i.), iprodione (98% a.i.), triazolone (97% a.i.), fludioxonil (95% a.i.), hymexazol (99.16% a.i.), trifloxystrobin (97% a.i.), and azoxystrobin (96% a.i.)], phenylaminopyrimidine fungicides [pyrimethanil (97% a.i.)] and methoxyacrylate fungicides [pyraclostrobin (97% a.i.)] were used to determine the sensitivity of a single pathogen strain under in vitro conditions (Table 1). Stock solutions of the active ingredient at 5000 µg/mL were produced by dissolving fungicides in acetone. Subsequently, the stock solutions were diluted with sterile distilled water and added into the PDA to obtain a range of concentrations, as presented in Table 2. PDA without fungicide served as the negative control. Mycelial discs (5 mm in diameter) were removed from the margins of 10-day-old cultures and transferred to the center of the solidified PDA plates treated with the different fungicide concentrations and the negative control. Three replicates of each treatment were conducted. When the control colony reached 3/4 of the size of the Petri dish, the colony’s diameter was measured in two perpendicular directions. The growth inhibition rates were calculated using the following formula: Inhibition rate of mycelial growth (%) = (Colony diameter of control group − colony diameter of experimental group)/(Colony diameter of control group − 5 mm) × 100%.

### 2.7. Data Analyses

The data for the biological characteristics were statistically analysed by one-way ANOVA using the Origin software package, version 2021 (OriginLab, USA). Mean values were compared using Duncan’s new multiple range test at the 5% (*p* < 0.05) significance level. The inhibiting rate of mycelial growth related to the control was calculated for all the fungicide concentrations. The median effective concentration (EC_50_) value was calculated by linear regressions of the inhibiting rates of mycelial growth versus the log_10_ transformation of the fungicide concentrations [13].

## 3. Results

### 3.1. Field Symptoms

The disease primarily affected the leaves of the sisal plants. More specifically, lesions mainly appeared in the middle and lower leaves of the plants. In the early stage of infection, dark reddish-brown, crescent- or semicircle- shaped lesions with distinct boundaries appeared at the leaf tips and margin (Figure 1A,B). As the disease developed, the disease lesions gradually expanded and enlarged, and at the same time, deepened in colour (Figure 1B,C). Severe depressions, as the tissues were prone to drying and consequent shrinkage, were also detected (Figure 1C). In the later stages, the lesions turned black-brown, connecting at the tips and margins of the leaves, and gradually expanded inward and downward (Figure 1B). Finally, the diseased leaves became desiccated and curled. In addition, the occurrence of MLBD varied between different seasons. In winter and spring (from December to the following April), the diseased field showed more severe symptoms. In contrast, the symptoms became nearly invisible in the autumn and winter (from May to November).

### 3.2. Pathogen Isolation and Pathogenicity Determination

**Figure 2:**
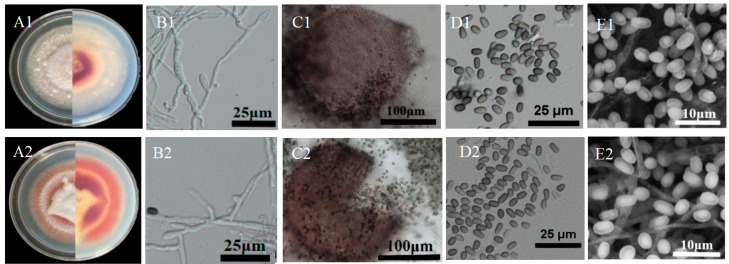
Morphological characteristics of pathogens. (**A**) The front (**left**) and back (**right**) of a colony; (**B**) mycelium; (**C**) pycnidium with an aperture or opening; (**D**) mature conidia under a light microscope; (**E**) mature conidia under scanning electron microscopy. The numbers 1 and 2 correspond to 22GX1-3 and 22GD1-4, respectively.

**Figure 3:**
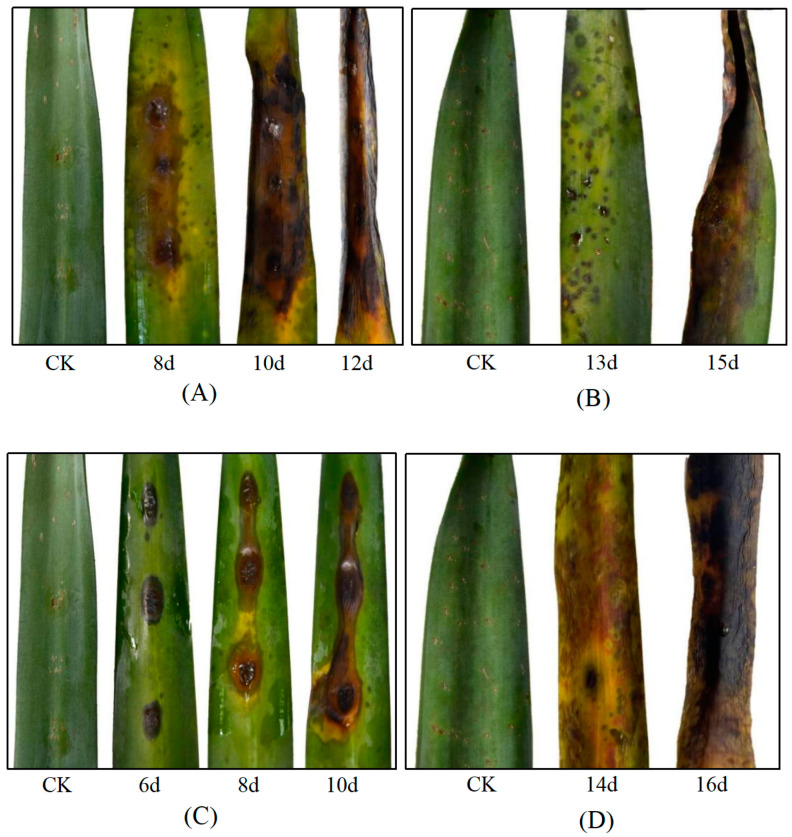
Symptoms of sisal leaves inoculated with pathogens. (**A**) Leaves inoculated with 22GX1-3 using mycelial plugs and (**B**) conidial suspension. (**C**) Leaves inoculated with 22GX1-4 using mycelial plugs and (**D**) conidial suspension.

A total of nine strains were obtained from the diseased leaf samples in Hongshan Farm and Dongfanghong Farm (Figure 2 and Figure S1). The pathogenicity tests indicated that sisal could be infected by two strains, including 22GX1-3 isolated from Hongshan Farm, and 22GD1-4 isolated from Dongfanghong Farm. Sisal leaves inoculated with 22GX1-3 and 22GD1-4 showed similar symptoms. Six days after placing mycelial plugs on the leaf surfaces, they developed dark brown spots at the inoculation site. As time went on, the lesion grew larger, its color changed to black-brown, and the leaves began to curl (Figure 3A,C). Thirteen days after spraying with a spore suspension, sisal leaves showed signs of infection, with lesions turning from reddish-brown to black-brown, eventually leading to leaf curling (Figure 3B,D). These lesions were similar to those observed on leaves in the field (Figure 1), while the control leaves remained symptomless (Figure S2). The same fungi were re-isolated from the diseased leaves, thereby satisfying Koch’s postulates.

### 3.3. Morphologic Characteristics

The colony morphology of 22GX1-3 and 22GD1-4 was similar (Figure 2), and both exhibited a slow mycelial growth rate. When cultured on PDA medium at 28 °C for 30 d, the colony diameter was 66~68 mm. The colonies were circular or irregular, and they was pale grey or grey-white, sometimes red; the mycelium often aggregated into droplet shapes on the surface; and the reverse side of the colony had orange-yellow pigmentation, and the mycelium around the periphery of the colony underside was usually colourless, gradually turning yellow towards the center (Figure 2(A1,A2)). Conidiophores were solitary and occasionally branched; immature conidia and conidiogenous cells were ellipsoidal to long ellipsoidal, hyaline, with round distal ends (Figure 2(B1,B2)). The pycnidium was spherical, brown to black, with an opening or aperture at its tip (Figure 2(C1,C2)). The mature conidia were pale brown to dark brown, unicellular, ellipsoidal, flattened, with thick walls, aseptate, and smooth-walled measuring 3.78–5.59 (mean = 4.62 ± 0.38) μm × 1.86–3.63 (mean = 2.58 ± 0.27) μm (Figure 2(D1,E1,D2,E2)). Based on the morphological characteristics of strains 22GX1-3 and 22GD1-4, it was determined that both strains belong to the same species of fungus. After comparison with related literature on *Phaeosphaeriopsis* spp., this fungus is similar to *Phaeosphaeriopsis* [26,27].

### 3.4. Sequence and Phylogenetic Analysis

**Figure 4:**
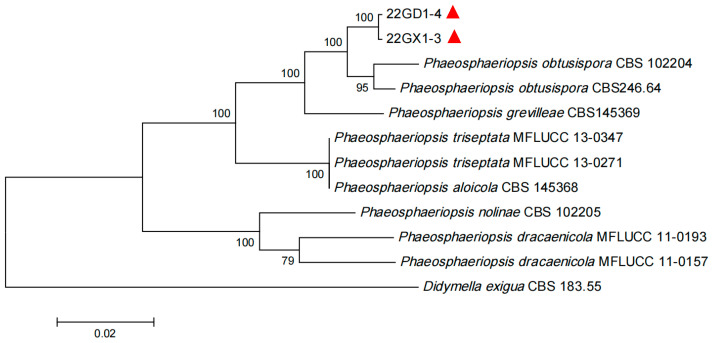
Phylogenetic tree inferred from concatenated ITS, *LSU*, and *RPB2* sequences using the neighbour-joining method. The bootstrap values (%) presented at the branches were calculated from 1000 replications. The values below 50% are hidden. The scale bar indicates a 5% sequence difference. The red triangle indicates the strains obtained in this study.

For pathogen identification, the total genomic DNA of these strains was used as a template for PCR amplification. Through cloning and sequencing of the PCR products, we obtained fragments of 593 bp (ITS), 929 bp (*LSU*), and 989 bp (*RPB2*) for 22GX1-3, and fragments of 593 bp (ITS), 936 bp (*LSU*), and 989 bp (*RPB2*) for 22GD1-4, as detailed in Table S1. A BLAST analysis showed that the similarity of fragments belonging to 22GX1-3 and 22GD1-4 with previously submitted *P. obtusispora* sequences (KJ396338, MK334366, and KP732017) was 99% to 100%. A phylogenetic tree was constructed using ITS, *LSU*, and *RPB2* sequences (Figure 4). 22GX1-3 and 22GD1-4 were clustered in the same evolutionary clade with *P. obtusispora* CBS 102204 and CBS246.64 with 100% bootstrap support. Thus, on the basis of the morphological and molecular characteristics, 22GX1-3 and 22GD1-4 isolated from diseased leaves of sisal plants in Guangxi and Guangdong provinces, China, were identified as *P. obtusispora*.

### 3.5. Biological Characterization of Phaeosphaeriopsis obtusispora

**Figure 5:**
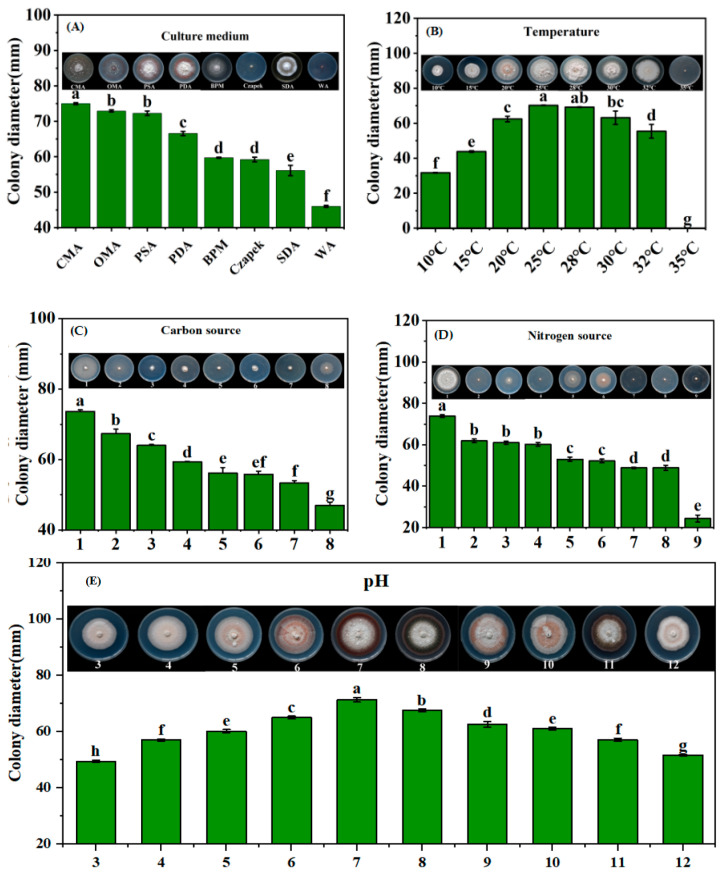
Biological characterization of *Phaeosphaeriopsis obtusispora*. (**A**) Culture medium; (**B**) temperature; (**C**) carbon source: 1. lactose; 2. D-sorbitol; 3. mannitol; 4. maltose; 5. fructose; 6. glucose; 7. xylose; 8. amidulin; (**D**) nitrogen source: 1. yeast extract powder; 2. aminocaproic acid; 3. isoleucine; 4. glycine; 5. peptone; 6. beef extract; 7. NH_4_Cl; 8. ammonia sulfate; 9. urea; (**E**) pH. Different letters indicate significant differences according to ANOVA.

The strain could grow on CMA, OMA, PSA, PDA, BPM, Czapek, SDA, and WA media (Figure 5A). The colony diameter was found to be the largest on CMA and OMA, averaging about 75 mm. However, the mycelium was relatively sparse in these two types of media. The diameters of colonies on PSA, PDA, BPM, Czapek, and SDA media were next, ranging between 56 and 66 mm. Among these, the mycelium on PSA, PDA, and SDA was more compact. The medium with the slowest mycelial growth rate was WA, with an average diameter of 46.00 mm. Therefore, PDA was considered as the most suitable medium for the growth of *P. obtusispora*, and it was used for the following experiments for the determination of the optimal growth conditions for the pathogen. When lactose was used as the carbon source, the mycelia were dense and grew the fastest, and the average colony diameter was 73.67 mm, whereas the utilization rate of amidulin by the strain was the lowest, and the average colony diameter was only 47.00 mm (Figure 5B). Additionally, when yeast extract powder was used as the nitrogen source, the mycelial densities were the greatest and grew at the fastest rate, and the average colony diameter was 73.83 mm; however, when urea was used as the nitrogen source, mycelia grew at the slowest rate, and the average colony diameter was only 24.33 mm, which was significantly smaller than colonies observed when other nitrogen sources were used (Figure 5C). The pathogen can grow in environments with temperatures ranging from 10 °C to 32 °C (Figure 5D). At 25 °C, the mycelium grew at the fastest rate, with an average colony diameter of 70.17 mm, making it the optimal growth temperature for this pathogen. At 28 °C, the average colony diameter was 69.00 mm; at 30 °C, the average colony diameter was 67.17 mm, indicating that temperatures between 25 °Cand 30 °C are suitable for the growth of this strain. However, at 35 °C, the growth of the mycelium dropped to 0 mm. The optimal pH for mycelial growth was 7.0, and the average colony diameter was 71.33 mm. The pathogen rapidly grew in environments with a pH range from 6.0 to 11.0. When the pH was below 5.0 or above 11.0, the growth of the mycelium was inhibited (Figure 5E).

### 3.6. Sensitivity of P. obtusispora Strain to Fungicides

Twelve conventional fungicides were screened for their antifungal activities against a single strain, 22GX1-3, of *P. obtusispora*. Interestingly, all twelve of the tested agents had inhibitory activities against the pathogen (Table 2). Among the twelve fungicides, difenoconazole was proven most effective in suppressing the mycelial growth of *P. obtusispora* (EC_50_ of 0.5380 µg/mL). This was followed by tebuconazole, myclobutanil, and pyraclostrobin, with EC_50_ values of 1.6835, 2.1218, and 15.7005 µg/mL for *P. obtusispora*, respectively. The antifungal activities of thiophanate-methyl, iprodione, triazolone, fludioxonil, hymexazol, trifloxystrobin, and pyrimethanil were lower, with EC_50_ values of 22.2619, 36.8578, 41.6926, 53.7377, 122.2286, 142.1241, and 192.2392 µg/mL for *P. obtusispora*, respectively. However, the antifungal activities of azoxystrobin were the lowest, with EC_50_ values of 667.5094 µg/mL for *P. obtusispora*.

## 4. Discussion

Sisal is an important tropical cash crop in China. Unfortunately, the emergence of numerous new diseases poses a significant threat to the development of the sisal industry [4,5,7,8,9]. The development of a correct diagnosis method is a fundamental requirement for effective disease management. In this work, through pathogen isolation and Koch’s postulates, two strains, namely 22GX1-3 and 22GD1-4, were considered as the causative pathogens of MLBD. Combined with morphological observation, it was found that the morphological characteristics of the two strains, 22GX1-3 and 22GD1-4, were similar to some species in the fungal genus *Phaeosphaeriopsis* spp. [26,27].

Currently, molecular biology techniques combined with the corresponding target genes are regarded as more accurate methods for identifying fungal species [28]. However, relying solely on a single target gene is often insufficient to distinguish all species within certain groups due to the significant genetic differences among fungi. Thus, multigene combined analysis has been used for resolution at the species level for higher reliability [28]. To this end, in this work, nine reference strains of the fungal genus *Phaeosphaeriopsis* were screened including sequences of ITS, *LSU*, and *RPB2*, and sequence similarity and genetic evolution analyses were conducted. The results showed that the similarity of fragments from 22GX1-3 and 22GD1-4 to previously submitted *P. obtusispora* sequences (KJ396338, MK334366, and KP732017) was 99% to 100%. Furthermore, the representative strains (22GX1-3 and 22GD1-4) clustered in the same evolutionary clade as *P. obtusispora* CBS 102204 and CBS 246.64 with 100% bootstrap support. Thus, through the BLASTn of sequence similarity and a phylogenetic tree using ITS-*LSU*-*RPB2* multigene combined analysis, the causative pathogens were identified as *P. obtusispora*. Undeniably, there are relatively few reference strains that could be used to establish genetic phylogenetic trees. In the future, there may be more reference strains of the fungal genus *Phaeosphaeriopsis* including the sequences of ITS-*LSU*-*RPB2,* and they could be used to make a further genetic evolution analysis.

The fungal genus *Phaeosphaeriopsis* spp. belongs to the family Paraphaeosphaeria. According to existing reports, it includes approximately twenty species (www.Indexfungorum.org, accessed on 22 March 2024) [29]. Currently, some related members of this genus have been reported in the literature to be plant pathogens associated with several diseases. For instance, *P. glaucopunctata* has been shown to cause leaf spot and necrosis on *Ruscus aculeatus* [30]; *P. musae* has been demonstrated to cause leaf spots on banana [31]; and *P. dracaenicola* has been proven to cause leaf spots on the leaves of *Dracaena lourieri* [27]. However, other members of this genus, mainly isolated from *Agave tequilana* [32], *Beaucarnea recurvata* [32], and *Ruscus aculeatus* [33], are saprobic fungi or plant endophytic fungi. The pathogen *P. obtusispora* was first reported as a saprobic fungi in the Agavaceae [29]. However, its plant-host diseases remained unreported. In this work, it was demonstrated that *P. obtusispora* can cause marginal leaf blight. Changes in agricultural and environmental conditions may affect pathogen pathogenicity and the frequency of infection. Sisal SMLB caused by *P. obtusispora* represents such a phenomenon and is considered as an emergent disease. To the best of our knowledge, this work reports for the first time the occurrence of marginal leaf blight on sisal plants caused by *P. obtusispora*.

Exploring the biological characteristics of pathogens aids in assessing how they adapt to various environmental conditions and in forecasting their potential to cause outbreaks in certain climates. In this work, *P. obtusispora* isolated from sisal plants grew fastest at 25 °C and pH 7. The highest utilization rates were achieved with lactose and yeast extract powder. The optimal medium for the growth of this strain might be PDA. The optimal growth temperature was 25 °C, and the stopping growth temperature was 35 °C. The temperature-related characteristics of *P. obtusispora* isolated in this work differed from those of other pathogens causing sisal disease. For example, *Neoscytalidium dimidiatum* and *Clonostachys rogersoniana,* which, respectively, cause canker disease [34] and leaf blight disease (unpublished) of sisal, grow vigorously in 10 °C-40 °C, whereas the strain that causes MLBD grows below 32 °C. Thus, the temperature from January to April in southern China provides favourable conditions for pathogen infection in the field, requiring attention when developing strategies to prevent and control disease. In addition, the optimum pH for the growth of *P. obtusispora* isolated in this work was similar to that of the strain causing canker disease of sisal [34], with both growing vigorously at a pH of 5 to 7. However, the strain causing marginal leaf blight grows vigorously at other values of pH (>7 or <5).

Fungicide screening is helpful for discovering chemicals agents that are effective against specific plant pathogens, especially in the absence of other effective disease management strategies. In this work, the bioassays showed that the selected twelve fungicides had different antifungal effects against a single strain, 22GX1-3, of *P. obtusispora*. Triazole fungicides are a class of broad-spectrum antifungal agents. Except for their inactivity against the Oomycetes within the phylum Stramenopiles, they are active against pathogens in the Ascomycota [15,35,36,37] and Basidiomycota [16,38,39]. Their mechanism of action involves affecting sterol biosynthesis, thereby disrupting the function of the fungal cell membrane [17]. The *Phaeosphaeriopsis* spp. in this work belong to Ascomycota, and the ten triazole fungicides examined had different impacts on controlling *P. obtusispora*. Particularly, difenoconazole exhibited the highest antifungal activities against *P. obtusispora* (EC_50_, 0.5380 µg/mL); other fungicides weakly inhibited the growth of *P. obtusispora*, in particular azoxystrobin (EC_50_, 667.5094 µg/mL). In addition, phenylaminopyrimidine fungicides such as pyraclostrobin, a mitochondrial respiratory inhibitor for pathogens in the Ascomycetes [18,40] and Oomycetes [19,41], and methoxyacrylate fungicides such as pyrimethanil, an infectious enzyme secretion inhibitor for plant pathogens such as *Venturia inaequalis*, *Alternaria solani*, and/or *Botrytis cinerea* [20,42,43], were also selected to test their antifungal activities against *P. obtusispora*. However, pyraclostrobin (EC_50_, 15.7005 µg/mL) and pyrimethanil (EC_50_, 192.2392 µg/mL) also weakly inhibited the growth of *P. obtusispora*. Thus, difenoconazole can be selected to control marginal leaf blight of sisal caused by *P. obtusispora*, while tebuconazole, myclobutanil, pyraclostrobin, hymexazol, trifloxystrobin, azoxystrobin, and pyrimethanil are not suitable for the control of *P. obtusispora*. Given that the results of laboratory bioassays do not always provide an accurate reflection of control efficacies in the field, field trials must be conducted. In addition, fungicide sensitivity may vary among strains within the same species. Thus, in this study, the sensitivity data based on a single strain, 22GX1-3, is still insufficient. In the future, more pathogen strains need to be collected from different planting areas and different years, and their fungicide sensitivity needs to be verified.

## 5. Conclusions

In sum, nine fungal strains were isolated from diseased leaves of sisal plants, and a new pathogen causing leaf disease of sisal was identified as *P. obtusispora*. The biological characteristics of *P. obtusispora* were clarified, and control agents against *P. obtusispora* were screened. Among the twelve fungicides, difenoconazole yielded the highest antifungal activity against a single strain, 22GX1-3, of *P. obtusispora*. Our data results provide a deep understanding of leaf disease MLBD in sisal and are anticipated to promote the development of efficient management strategies for controlling leaf diseases of sisal caused by *P. obtusispora*.

## Figures and Tables

**Table 1:** Fungicides and experimental concentration gradients.

Fungicides	Manufacturer	Concentration Gradient (µg/mL)
Hymexazol (99.16% a.i.)	Jiangsu Heye Agrochemical Co., Ltd., Yancheng, China	80, 60, 40, 20, 10
Iprodione (98% a.i.)	Hubei Wanye Medicine Co., Ltd., Wuhan, China	10, 5, 1, 0.5, 0.05
Myclobutanil (96% a.i.)	Hubei Supuer Chemical Co., Ltd., Wuhan, China	15, 7.5, 1, 0.5, 0.1
Pyraclostrobin (97% a.i.)	Nanjing Gaozheng Agricultural Chemical Co., Ltd., Nanjing, China	15, 10, 1, 0.5, 0.05
Triazolone (97% a.i.)	Jiangsu Arrow Agrochemical Co., Ltd., Yancheng, China	80, 60, 40, 20, 10
Thiophanate-Methyl (95% a.i.)	Shanghai Yuanye Biology Science and Technology Co., Ltd., Shanghai, China	10, 8, 1, 0.8, 0.4
Difenoconazole (96% a.i.)	Beijing Green Nonghua Plant Protection Technology Co., Ltd., Beijing, China	10, 1, 0.1, 0.01, 0.001
Azoxystrobin (96% a.i.)	Shang Hai De Mo Hua Xue Technology Co., Ltd., Shanghai, China	15, 10, 1, 0.1, 0.01
Fludioxonil (95% a.i.)	Hebei Xingbai Agriculture Technology Co., Ltd., Shijiazhuang, China	20, 10, 1, 0.5, 0.1
Tebuconazole (97.1% a.i.)	Jiangsu Fengdeng Crop Protection Co., Ltd., Changzhou, China	5, 1.5, 0.5, 0.3, 0.15
Pyrimethanil (97% a.i.)	Jiangxi Zhongxun Agrochemical Co., Ltd., Nanchang, China	50, 25, 10, 1, 0.5
Trifloxystrobin (97% a.i.)	Bayer (China) Co., Ltd., Shanghai, China	20, 10, 1, 0.5, 0.1, 0.05

**Table 2:** Toxicity of twelve fungicides against mycelial growth of *Phaeosphaeriopsis obtusispora* strain 22GX1-3.

Fungicide	Regression Equation	Correlation Coefficient (r)	EC_50_^a^/µg/mL
Difenoconazole	y = 0.5929x + 5.1596	0.9483	0.5380
Tebuconazole	y = 1.8915x + 4.5722	0.9886	1.6835
Myclobutanil	y = 1.1636x + 4.6198	0.9939	2.1218
Pyraclostrobin	y = 0.6366x + 4.2386	0.9864	15.7005
Thiophanate-Methyl	y = 0.8889x + 3.8021	0.9759	22.2619
Iprodione	y = 0.4872x + 4.2368	0.9869	36.8578
Triazolone	y = 1.1520x + 3.1337	0.9856	41.6926
Fludioxonil	y = 0.3898x + 4.3256	0.9742	53.7377
Hymexazol	y = 1.3385x + 2.2064	0.9839	122.2286
Trifloxystrobin	y = 0.3823x + 4.1771	0.9838	142.1241
Pyrimethanil	y = 0.7482x + 3.2913	0.9770	192.2392
Azoxystrobin	y = 0.3002x + 4.1523	0.9800	667.5094

^a^ EC_50_ = median effective concentration.

## Data Availability

Sequence data from this article can be found in GenBank at https://www.ncbi.nlm.nih.gov/genbank/ (accessed on 5 May 2024) with the accession numbers listed in the Results. All other relevant data are within the paper.

## Supplementary Materials

The following supporting information can be downloaded at: https://www.mdpi.com/article/10.3390/jof10070486/s1, Figure S1. Upper and lower surface of non-pathogenic bacteria (A–H) obtained from diseased leaf tissues of sisal plants; Figure S2. Pathogenicity test of non-pathogenic bacteria inoculated by placing fungus PDA plugs for 12 d. The figures A-H in Figure S2 correspond to the non-pathogenic bacteria (A–H) one by one in Figure S1. CK was the negative control that was inoculated with sterile PDA plugs (5 mm in diameter); Table S1. The ITS, *LUS* and *RPB2* sequences of *Phaeosphaeriopsis obtusispora* amplified using the primer ITS1/ITS4 [22], LROR(F)/LR5(R) [23], and fRPB2-5F (F)/fRPB2-7cR [24]; Table S2. Formulation of the media.
