# Supplementary material for: Characterization and Fungicide Sensitivity of Phaeosphaeriopsis obtusispora That Causes Marginal Leaf Blight in Agave hybrid H.11648
Source: J Fungi (Basel). 2024 Jul 14;10(7):486. doi: 10.3390/jof10070486 (PMC11278330; doi:10.3390/jof10070486)
Supplement: Supplementary file 1 [file jof-10-00486-s001.zip › Figure S1.pdf]

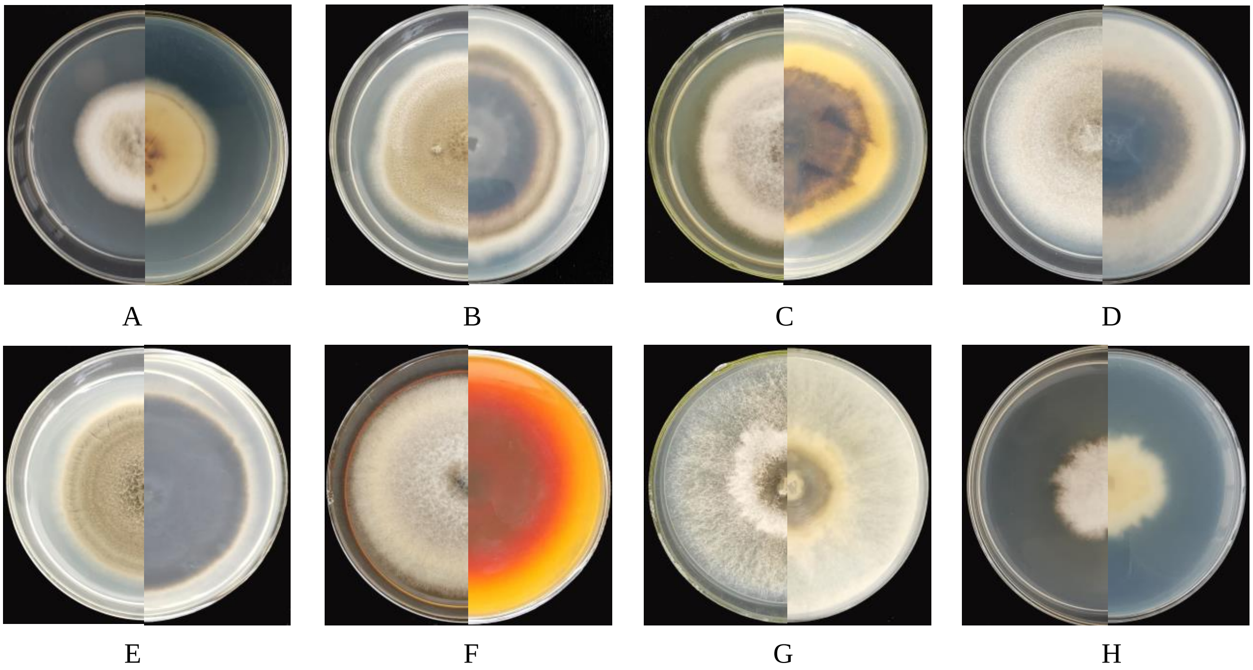

Figue S1. The upper and lower surface of Non-pathogenic strains (A-H) obtained from diseased leaf tissues of sisal plants.
