# Supplementary material for: Characterization and Fungicide Sensitivity of Phaeosphaeriopsis obtusispora That Causes Marginal Leaf Blight in Agave hybrid H.11648
Source: J Fungi (Basel). 2024 Jul 14;10(7):486. doi: 10.3390/jof10070486 (PMC11278330; doi:10.3390/jof10070486)
Supplement: Supplementary file 1 [file jof-10-00486-s001.zip › Figure S2 .pdf]

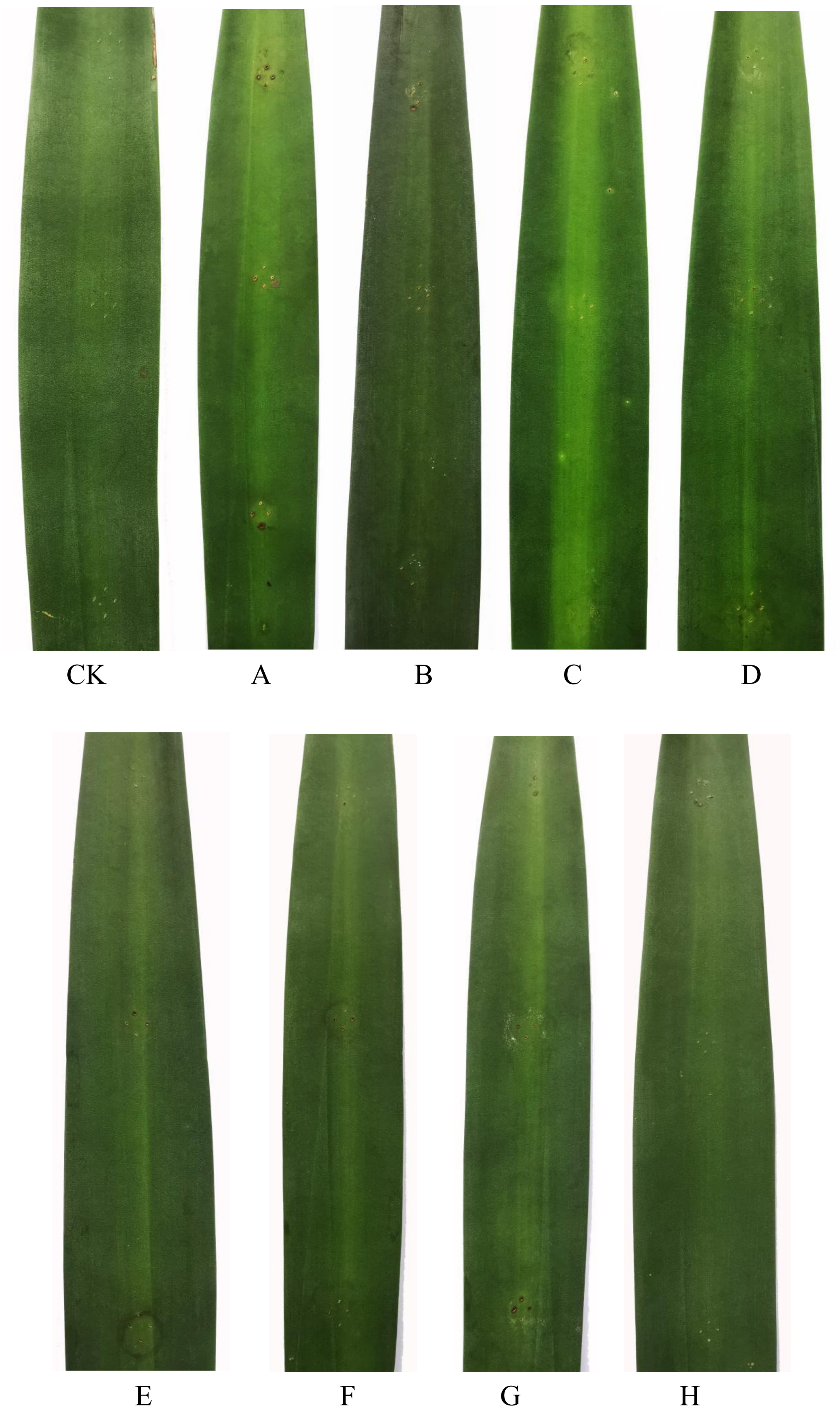

Figure S2. Pathogenicity test of Non-pathogenic strains inoculated by placing fungus PDA plugs for 12d. The figures of A-H in Figure S2 corresponds to the non-pathogenic strains (A-H) one by one in Figure S1. CK was the negative control that was inoculated with sterile PDA plugs (5 mm in diameter).
