# Supplementary material for: Characterization and Fungicide Sensitivity of Phaeosphaeriopsis obtusispora That Causes Marginal Leaf Blight in Agave hybrid H.11648
Source: J Fungi (Basel). 2024 Jul 14;10(7):486. doi: 10.3390/jof10070486 (PMC11278330; doi:10.3390/jof10070486)
Supplement: Supplementary file 1 [file jof-10-00486-s001.zip › Table S1.pdf]

Table S1. The ITS, *LUS* and *RPB2* sequences of *Phaeosphaeriopsis obtusispora* amplified using the primer ITS1/ITS4[22], LROR(F)/LR5(R)[23], and fRPB2-5F (F)/fRPB2-7cR [24].

No.	Gene	Isolate	Sequences
1	ITS (593bp)	GX1-3	TCCTCCGCTTATTGATATGCTTAAGTTCAGCGGGTATCCCTACCTGATCCGA<br>GGTCAAAAGTTAAAAAATAGCTTAATGGATACCACTGTTTATTAGCTGGG<br>GCTGCAAATTGTGCTGCGCTCCAATACCAATACAATGGCTGCCAATTATTT<br>TAAGGCGAGTCCAAACATGATGAGAGGACAAACACCCAACACCAAGCAG<br>AGCTTGAAGGTACAAATGACGCTCGAACAGGCATGCCCCATGGAATACCA<br>AGGGGCGCAATGTGCGTTCAAAGATTCGATGATTCACTGAATTCTGCAATT<br>CACACTACTTATCGCATTTCGCTGCGTTCTTCATCGATGCCAGAACCAAGA<br>GATCCGTTGTTGAAAGTTGTAATTATTATAATTATTTTAGACGCTGAATAA<br>AAATTAAAAAGGTTGTATATTTGTCCAGTTGGTGGGCGAGCCCACCAAGG<br>AAACATAAGTACTCAAAAGACAAGGATGGAGAAAGGGAGTTTACTCGTG<br>ATCCAGCGATGAAACTGGGACTTTCCACTCCCCCAAAATCAGTAGAAAAC<br>TACTGTGTTTAATGATCCTTCCGCAGGTTCACCTACGGA
2	*LUS* (929bp)	GX1-3	ACCCGCTGAACTTAAGCATATCAATAAGCGGAGGAAAAGAAACCAACAG<br>GGATTGCCCTAGTAACGGCGAGTGAAGCGGCAACAGCTCAAATTTGAAAT<br>CTGGCTCTTTTAGAGTCCGAGTTGTAATTTGCAGAGGGCGCTTTGGCATTG<br>GCAGCGGTCCAAGTTCTTTGGAACAGGACGTCACAGAGGGTGAGAATCCC<br>GTACGTGGTCGCTAGCCTTCGCCGTGTAAAGCCCCTTCGACGAGTCGAGTT<br>GTTTGGGAATGCAGCTCTAAATGGGAGGTAAATTTCTTCTAAAGCTAAATA<br>CTGGCCAGAGACCGATAGCGCACAAGTAGAGTGATCGAAAGATGAAAAG<br>CACTTTGGAAAGAGAGTCAAATAGCACGTGAAATTGTTGAAAGGGAAGC<br>GCTTGCAGCCAGACTTGCCTGTAGTTGCTTATCTGGACTTTTGTCCAGTGCA<br>CTCTTCTGCGGGCAGGCCAGCATCAGTTTGGGCGGTTGGATAAAGGTCTCT<br>GTCATGTACCTCTCTTCGGGGAGGCCTTATAGGGGAGACGACATGCAACC<br>AGCCTGGACTGAGGTCCGCGCATTTGCTAGGATGCTGGCGTAATGGCTGTA<br>AGCGGCCCGTCTTGAAACACGGACCAAGGAGTCTAACATCTATGCGAGTG<br>TTTGGGTGTCAAGCCCAGACGCGTAATGAAAGTGAACGGAGGTGGGAAC<br>CCTTTGGGTGCACCATCGACCGATCCTGATGTCTTCGGAAAGATTTGAGTA<br>AGAGCATGGCTGTGGGACCCGAAAGATGGTGAACTATGCTTGAATAGGGT<br>GAAGCAGAGGAACTCTGGTGGAGGCTCGCAGCGGTTCTGACGTGCAATCG<br>ATCGTCAATTTGGGCATAGGGGCGAAAGACTATCGACTATCTAGTAGCTG<br>GTTCCTGCCGAAGTTTCCCTCAGGA

3	*RPB2*<br>(989bp)	GX1-3	CCCATAGCTTGTTTGCCCATGGCAGATTGGTAAGTATTACGGGGTGACTGAT<br>TGTGATCCGGGAAAGGAATGATACTGGCACAAATACCGAGAATCATAGCA<br>GGATGGATCTCGCAATGAGTGTAGGCATGGATGCGTGGGTCAGGCTTAGGC<br>TTAATGCGCTTGAGACGATCCTTGCCAAGAGCCGCTCGCTCATTAGAAGGC<br>AGGCCTAACTTCATGCCTCGCCACTCCTCAAGATCCTCTGGGGAGAATGTG<br>ATCATAGCAGTCTCTTCTTCCTCGGCGTCGAGGTATTCGATCACACCGTTTT<br>GGATTAGGCCCTTCCATCCATAAGTGTGTTCTGCAATATCTTCTTCGCTCCA<br>ACCTGCGCGATTATCAGACTCTAGCTGTTCTTCCACGAGCTTATTGCTTATTG<br>CTCGATCGAAGACGAGCTCATTGCGGTTGGGCTTCCGAGGATCGGTTTCGA<br>CAACGAATAGAGGTCGCATAACACGACCAGCATCGGTAAAGATCTTGAAT<br>TCTCGATCACGAATATCGCGAATCAAACTCATCTCGTATGATAGAGTTCCGT<br>TTCTTCGCAGTTCTTGAACGACAGAGACCAGTTGTTGCGCGTTAGAGTGGA<br>CACCAACCCAAACGCCATTGACAAAGACTTTAGTAGCCTCGGGGTTCTGAT<br>TCTGATCGTACTCCTCCAGAAGCTGCATGTTGCGCTGTGACATGAAGTCAA<br>TGATGGGCGTGGCGTCACTGCCGACGCTGACGTAGCACATCAAAGACAGA<br>TTCTTGACTAAGCCGCAGGGCTGGTCTTCTGGGGTTTCGGCAGGGCATACG<br>AGGCCCCAATGAGAGTTATGTAATTGTCGTGGCTTGGCCAGCTTACCATCG<br>CGCCCGACGGGAGTATTCGTTCGCCTGAGATGGGATAGGGTGGACGCATAC<br>GTGTAGCGATTCAACACTTGTGAGACGCCAGCCTTTGCGGACGCGGCCTTC<br>TTCTGATCACCCCAATC
4	ITS<br>(593bp)	GD1-4	TCCGTAGGTGAACCTGCGGAAGGATCATTAAACACAGTAGTTTTCTACTGA<br>TTTTGGGGGAGTGGAAAGTCCCAGTTTCATCGCTGGATCACGAGTAAACTC<br>CCTTTCTCCATCCTTGTCTTTTGAGTACTTATGTTTCCTTGGTGGGCTCGCCC<br>ACCAACTGGACAAATATACAACCTTTTTAATTTTTATTCAGCGTCTAAAATA<br>ATTATAATAATTACAACTTTCAACAACGGATCTCTTGGTTCTGGCATCGATG<br>AAGAACGCAGCGAAATGCGATAAGTAGTGTGAATTGCAGAATTCAGTGAA<br>TCATCGAATCTTTGAACGCACATTGCGCCCCTTGGTATTCCATGGGGCATGC<br>CTGTTCGAGCGTCATTTGTACCTTCAAGCTCTGCTTGGTGTTGGGTGTTTGTC<br>CTCTCATCATGTTTGGACTCGCCTTAAAATAATTGGCAGCCATTGTATTGGTA<br>TTGGAGCGCAGCACAATTTGCAGCCCCAGCTAATAAACAGTGGTATCCATT<br>AAGCTATTTTTTAACTTTTGACCTCGGATCAGGTAGGGATACCCGCTGAACT<br>TAAGCATATCAATAAGCGGAGGA

5	*LUS*<br>(936bp)	GD1-4	ACCCGCTGAACTTAAGCATATCAATAAGCGGAGGAAAAGAAACCAACAG<br>GGATTGCCCTAGTAACGGCGAGTGAAGCGGCAACAGCTCAAATTTGAAAT<br>CTGGCTCTTTTAGAGTCCGAGTTGTAATTTGCAGAGGGCGCTTTGGCATTGG<br>CAGCGGTCCAAGTTCTTTGGAACAGGACGTCACAGAGGGTGAGAATCCCG<br>TACGTGGTCGCTAGCCTTCGCCGTGTAAAGCCCCTTCGACGAGTCGAGTTG<br>TTTGGGAATGCAGCTCTAAATGGGAGGTAAATTTCTTCTAAAGCTAAATACT<br>GGCCAGAGACCGATAGCGCACAAGTAGAGTGATCGAAAGATGAAAAGCA<br>CTTTGGAAAGAGAGTCAAATAGCACGTGAAATTGTTGAAAGGGAAGCGCT<br>TGCAGCCAGACTTGCCTGTAGTTGCTTATCTGGACTTTTGTCCAGTGCACTC<br>TTCTGCGGGCAGGCCAGCATCAGTTTGGGCGGTTGGATAAAGGTCTCTGTC<br>ATGTACCTCTCTTCGGGGAGGCCTTATAGGGGAGACGACATGCAACCAGCC<br>TGGACTGAGGTCCGCGCATTTGCTAGGATGCTGGCGTAATGGCTGTAAGCG<br>GCCCGTCTTGAAACACGGACCAAGGAGTCTAACATCTATGCGAGTGTTTGG<br>GTGTCAAGCCCAGACGCGTAATGAAAGTGAACGGAGGTGGGAACCCTTTG<br>GGTGCACCATCGACCGATCCTGATGTCTTCGGAAGGATTTGAGTAAGAGCA<br>TGGCCGTTGGGACCCGAAAGATGGTGAACTATGCTTGAATAGGGTGAAGC<br>CAGAGGAAACTCTGGTGGAGGCTCGCAGCGGTTCTGACGTGCAAATCGAT<br>CGTCAAATTTGGGCATAGGGGCGAAAGACTAATCGAACTATCTAGTAGCTG<br>GTTCCTGCCGAAGTTTCCCTCAGGA
6	*RPB2*<br>(989bp)	GD1-4	GATTGGGGTGATCAGAAGAAGGCCGCGTCCGCAAAGGCTGGCGTCTCACA<br>AGTGTCCAATCGCTACACGTATGCGTCCACCCTATCCCATCTCAGGCGAAC<br>GAATACTCCCGTCGGGCGCGATGGTAAGCTGGCCAAGCCACGACAATTAC<br>ATAACTCTCATTGGGGCCTCGTATGCCCTGCCGAAACCCCAGAAGGCCAG<br>GCCTGCGGCTTAGTCAAGAATCTGTCTTTGATGTGCTACGTCAGCGTCGGC<br>AGTGACGCCACGCCCATCATTGACTTCATGTCACAGCGCAACATGCAGCT<br>TCTGGAGGAGTACGATCAGAATCAGAACCCCGAGGCTACTAAAGTCTTTG<br>TCAATGGCGTTTGGGTTGGTGTCCACTCTAACGCGCAACAACTGGTCTCTG<br>TCGTTCAAGAACTGCGAAGAAACGGAACTCTATCATACGAGATGAGTTTG<br>ATTCGCGATATTCGTGATCGAGAATTCAAGATCTTTACCGATGCTGGTCGT<br>GTTATGCGACCTCTATTCGTTGTCGAAACCGATCCTCGGAAGCCCAACCGC<br>AATGAGCTCGTCTTCGATCGAGCAATAAGCAATAAGCTCGTGGAAGAACA<br>GCTAGAGTCTGATAATCGCGCAGGTTGGAGCGAAGAAGATATTGCAGAAC<br>ACACTTATGGATGGAAGGGCCTAATCCAAAACGGTGTGATCGAATACCTC<br>GACGCCGAGGAAGAAGAGACTGCTATGATCACATTCTCCCCAGAGGATCT<br>TGAGGAGTGGCGAGGCATGAAGTTAGGCCTGCCTTCTAATGAGCGAGCGG<br>CTCTTGGCAAGGATCGTCTCAAGCGCATTAAGCCTAAGCCTGACCCACGC<br>ATCCATGCCTACACTCATTGCGAGATCCATCCTGCTATGATTCTCGGTATTT<br>GTGCCAGTATCATTCCTTTCCCGGATCACAATCAGTCACCCCGTAATACTT<br>ACCAATCTGCCATGGGCAAACAAGCTATGGG
