# Supplementary material for: Characterization and Fungicide Sensitivity of Phaeosphaeriopsis obtusispora That Causes Marginal Leaf Blight in Agave hybrid H.11648
Source: J Fungi (Basel). 2024 Jul 14;10(7):486. doi: 10.3390/jof10070486 (PMC11278330; doi:10.3390/jof10070486)
Supplement: Supplementary file 1 [file jof-10-00486-s001.zip › Table S2.pdf]

Table S2. Formulation of the media.

Medium	Formulation
PDA	peeled potato, 200 g; glucose, 20 g; agar powder, 20 g, distilled water to 1 L
CMA	corn flour, 20 g; agar powder, 18 g, distilled water to 1 L
OMA	oat flour, 20g; agar powder, 18 g, distilled water to 1 L
PSA	peeled potato, 200 g; sucrose, 20 g; agar powder, 20 g, distilled water to 1 L
Cazpek	sucrose, 20 g; NaNO3, 3g; KCl, 0.5g; K2HPO4, 1g; MgSO4, 0.5g; FeSO4, 0.01g; agar powder, 20 g, distilled water to 1 L
BMP	peptone, 10g; Beef extract, 3g; NaCl, 5g; agar powder, 20 g, distilled water to 1 L
SAB	glucose, 40g; Yeast extract powder, 10g; peptone, 10g;agar powder, 20 g, distilled water to 1 L
WA	agar powder, 20 g, distilled water to 1 L
